# Microbial Synthesis of (*S*)- and (*R*)-Benzoin in Enantioselective Desymmetrization and Deracemization Catalyzed by *Aureobasidium pullulans* Included in the Blossom Protect™ Agent

**DOI:** 10.3390/molecules26061578

**Published:** 2021-03-12

**Authors:** Renata Kołodziejska, Renata Studzińska, Agnieszka Tafelska-Kaczmarek, Hanna Pawluk, Dominika Mlicka, Alina Woźniak

**Affiliations:** 1 Department of Medical Biology and Biochemistry, Faculty of Medicine, Nicolaus Copernicus University in Toruń, Collegium Medicum in Bydgoszcz, 24 Karłowicz Street, 85-092 Bydgoszcz, Poland; hannapawluk1@wp.pl (H.P.); mikaa0901@gmail.com (D.M.); alina-wozniak@wp.pl (A.W.); 2 Department of Organic Chemistry, Faculty of Pharmacy, Nicolaus Copernicus University in Toruń, Collegium Medicum in Bydgoszcz, 2 Jurasz Street, 85-089 Bydgoszcz, Poland; rstud@cm.umk.pl; 3 Department of Organic Chemistry, Faculty of Chemistry, Nicolaus Copernicus University in Toruń, 7 Gagarin Street, 87-100 Toruń, Poland; tafel@umk.pl

**Keywords:** desymmetrization, bioreduction, microbial synthesis, asymmetric, microorganism, α-hydroxy ketones

## Abstract

In this study, we examined the *Aureobasidium pullulans* strains DSM 14940 and DSM 14941 included in the Blossom Protect™ agent to be used in the bioreduction reaction of a symmetrical dicarbonyl compound. Both chiral 2-hydroxy-1,2-diphenylethanone antipodes were obtained with a high enantiomeric purity. Mild conditions (phosphate buffer [pH 7.0, 7.2], 30 °C) were successfully employed in the synthesis of (*S*)-benzoin using two different methodologies: benzyl desymmetrization and *rac*-benzoin deracemization. Bioreduction carried out with higher reagent concentrations, lower pH values and prolonged reaction time, and in the presence of additives, enabled enrichment of the reaction mixture with (*R*)-benzoin. The described procedure is a potentially useful tool in the synthesis of chiral building blocks with a defined configuration in a simple and economical process with a lower environmental impact, enabling one-pot biotransformation.

## 1. Introduction

Derivatives of α-hydroxy ketones play an important role in asymmetric organic synthesis. They are extremely valuable components of more architecturally complex compounds, offering a wide spectrum of applications. Optically active α-hydroxy ketones (also called acyloins) are considered useful building blocks in the preparation of various pharmaceuticals and chemicals thanks to their functional properties. Synthetic α-hydroxy ketones with the *R* configuration are used as precursors in the synthesis of some antidepressants (bupropion), selective inhibitors of amyloid-β protein production used in the treatment of Alzheimer’s disease, and as inducers of apoptosis in oral tumors and antifungal agents [1,2,3]. Acyloins are also found as structural subunits of natural compounds used in pharmacotherapy, for example in kurasoins A and B, protein farnesyltransferase inhibitors produced by *Penicillium sp.* FO-3929 [4], and in natural antitumor antibiotics, such as chromomycin A3, epothilones, mithramycin and olivomycin A [5,6].

1,2-Diaryl-2-hydroxyethanone structures (benzoins) are useful as urease inhibitors [2], as building blocks for the synthesis of heterocycles and multifunctional compounds such as diols, diamines, amino alcohols and epoxides [7,8,9].

There are many strategies, both chemical and biochemical, leading to the synthesis of enantiomerically enriched α-hydroxy ketones. Hydrobenzoins may be prepared by chemical means using oxidation of prochiral enolates in a reaction catalyzed by chiral oxidants, dihydroxylation of silyl enol ethers, RuO_4_-catalyzed reaction of ketohydroxylation of olefins, mono-oxidation of diols using a chiral catalyst; e.g., copper, or oxidation of racemic benzoins by a chiral iron or cobalt complex [10,11,12,13,14,15,16,17]. In addition, α-hydroxyketones can be obtained by direct α-oxygenation of ketones in the presence of chiral amino acids (l-proline, l-alanine) and by benzoin condensation catalyzed by chiral thiazolium or triazolium salts (chiral metallophosphites) [18,19,20,21,22]. Other strategies leading to formation of optically active benzoins involve biocatalysis, which uses natural tools–catalytic proteins. For instance, thiamine diphosphate-dependent lyases (ThDP-lyases) catalyze the umpolung carboligation of aldehydes to yield chiral α-hydroxy ketones. In addition to ThDP-lyases employed as bioreagents, catalysts, such as pyruvate decarboxylase (PDC), benzoylformate decarboxylase (BFD), and benzaldehyde lyase (BAL) [23] are included. Hydrolase-catalyzed kinetic resolutions or chemo-enzymatic dynamic kinetic resolutions of racemate have proven to be an efficient method of obtaining enantiopure α-hydroxy ketones. Several kinetic resolutions of racemic hydrobenzoins have been described using different lipases and esterases [24]. Enantiomerically enriched α-hydroxy ketones can also be successfully produced using the catalytic properties of oxidoreductases in the following reactions: reduction of diketones, oxidation of vicinal diols and deracemization (stereoinversion) of hydroxy ketones [25,26].

Biotransformation based on the reduction reaction of symmetrical diaryl diketones is carried out mainly in the presence of whole cells of microorganisms or isolated oxidoreductases. Biocatalytic reduction of benzils to chiral benzoin has been achieved by using different microorganisms, leading to the desired α-hydroxy ketone. In some cases, the reaction continues and yields the corresponding α-diol. The first attempts at microbiological reduction of benzil to benzoin were carried out with low or moderate selectivity [27,28,29]. *Saccharomyces cerevisiae* has been used for the production of benzoin with the *R* configuration with an enantiomeric excess (ee) of up to 50%, while reduction of other derivatives, 4-Me-Ph and furyl, was achieved with 36% and 82% ee, respectively [28]. Benzil and a series of *para*-substituted benzils have been reduced using *Cryptococcus macerans* to obtain (*S*)-benzoins with enantiomeric excesses of 20–30% [29]. Selection of strains of microorganisms has had a significant impact on the enantioselectivity of symmetrical diaryl diketone bioreduction. T. Saito et al. tested several different *Bacillus cereus* (bacterial) strains towards the selective reduction of a diaryl diketone. Of the strains examined, only wild-type *Bacillus cereus* Tim-r01 selectively reduced benzil to (*S*)-benzoin with a high 94% ee (92% yield) [30]. A. S. Demir et al. evaluated the ability of four different species of fungi (*Rhizopus oryzae* (ATCC 9363), *Rhizopus oryzae* (72465), *Rhizomucor miehei* (72460) and *Rhizomucor pusillus* (72561)) to convert benzil into benzoin and hydrobenzoin. *Rhizopus oryzae* (ATCC 9363) allows obtaining (*R*)-benzoin with >99% ee, while *Rhizomucor pusillus* (72561) yielded (*S*)-benzoin with 73% ee [31]. J. Konishi et al. obtained optically pure (*R*)-benzoin using *Xanthomonas oryzae* IAM 1657, which was selected via screening tests from multiple types of cultures [32]. Another group of fungi tested for selective properties in the biotransformation of benzils were *Aspergillus oryzae* and *Fusarium roseum* strains. Of the strains examined, *Aspergillus oryzae* OUT5048 and *Fusarium roseum* OUT4019 were found to be effective biocatalysts. Benzil and *ortho*-, *meta*-, and *para*-substituted derivatives of benzil were reduced to their corresponding benzoins by *A. oryzae* OUT5048 with up to 94% ee (benzil 94% ee) and by *F. roseum* OUT4019 with up to 98% ee (benzil 95% ee) [7]. Monoreduction of different 1,2-diaryl-1,2-diketones was carried out in a reaction catalyzed by lyophilized whole cells of *Pichia glucozyma* CBS 5766, selected among the following yeasts tested: *Pichia minuta* CBS 1708, *Pichia fermentans* DPVPG 2770, *Pichia glucozyma* CBS 5766, *Pichia etchellsii* CBS 2011, *Candida utilis* CBS 621 and *Kluyveromyces marxianus* CBS 397. The optical yield of benzoins from symmetrical benzils was 54–99% ee, and 1,2-diphenylethanone was reduced with a 75% enantiomeric excess and 99% conversion [9]. Using the same microorganism *P. glucozyma* CBS 5766, Maria Caterina (M.C). Fragnelli et al. successfully conducted benzil biotransformation in water/organic solvent two-liquid-phase systems. An increase in enantioselectivity was observed: use of *n*-heptane:water at 1:1 *v*/*v* yielded (*S*)-benzoin with a 90% conversion and 99% ee. The presence of the organic solvent influences the redox systems implicated in the reactions, preventing the formation of the corresponding diols. Under optimized conditions, other diaryl benzoins were obtained with 85–99% ee [33]. Oda et al. conducted asymmetric reduction of benzil to (*S*)-benzoin by *Penicillium claviforme* IAM 7294 in a two-phase system in a liquid–liquid interface bioreactor (L-L IBR) using a unique polymeric material, ballooned microsphere (MS). The L-L IBR showed superior performance, yielding 14.4 g/L-organic phase of (*S*)-benzoin (99.0% ee) [34].

Reduction of diketones with isolated enzymes is definitely less popular due to the need for expensive cofactors. R. Maruyama et al. used isolated recombinant benzil reductase from live *B. cereus* cells overexpressed in *Escherichia coli*. *B. cereus* benzil reductase enables selective reduction of the distal ketone group to a benzene ring, yielding optically pure (*S*)-benzoin [35].

Herein we report selective desymmetrization of symmetrical diaryl diketones using the antifungal agent Blossom Protect™. Previously, we described an effective biotransformation of prochiral sp^2^ hybridized compounds using the Boni Protect preparation as a catalyst. Chiral secondary alcohols and α- and β-hydroxy esters were obtained with high optical purity [36,37,38,39].

## 2. Results and Discussion

Several approaches to asymmetric biotransformation aiming to obtain high optical purity products are available. One can select the optimal bioreagent among several tested to perform the desired biotransformation with a high ee or to modulate the conditions of a reaction catalyzed by a microorganism yielding a moderate ee so as to improve the optical purity of the product. Optimization of the bioprocess parameters is one of the strategies affecting the enantioselectivity of biotransformation alongside site-directed-mutagenesis. Conversions and stereoselectivity can be improved by using enzyme inhibitors (high concentration of substrate, additives), thermal deactivation (temperature effect on selectivity) and organic cosolvents.

**Scheme 1:**
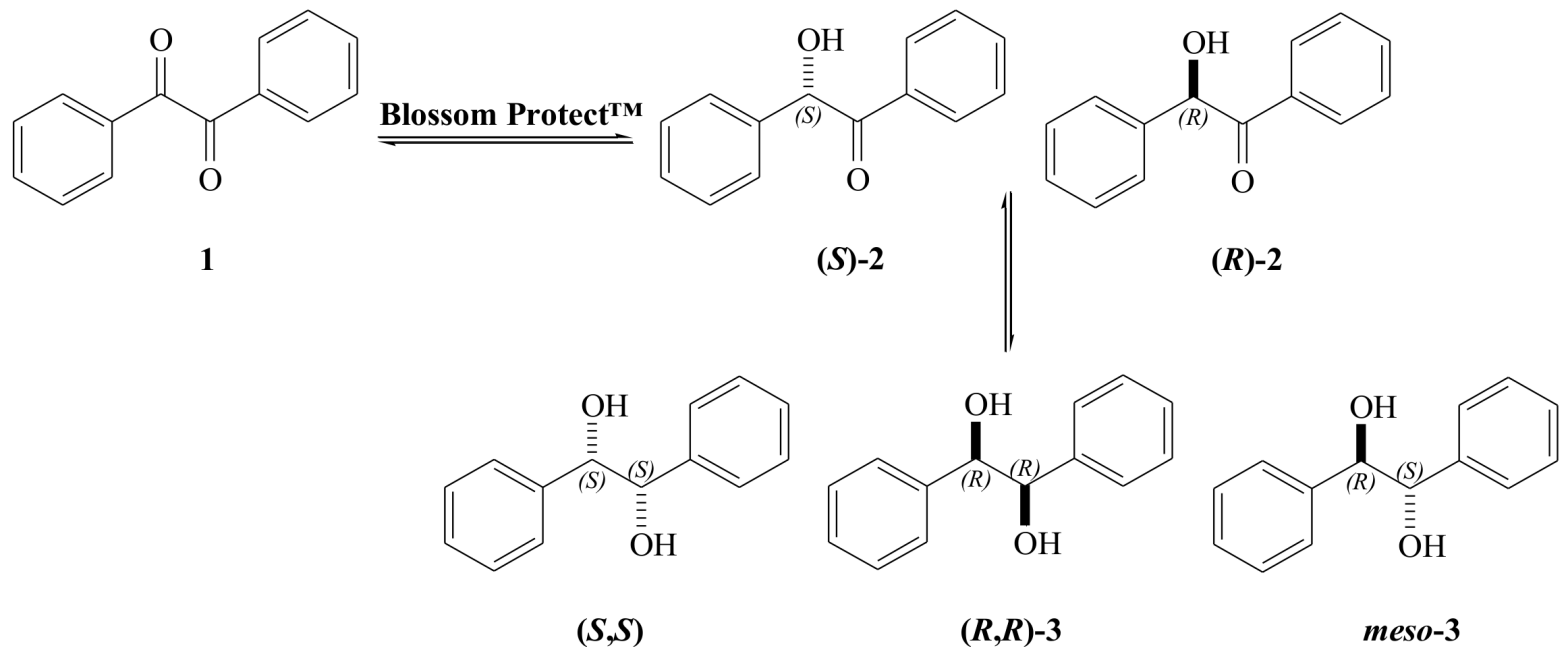
Biotransformation of a symmetrical diketone catalyzed by Blossom Protect™ containing live *A. pullulans* cells.

*Aureobasidium pullulans* strains DSM 14940 and DSM 14941 included in the Blossom Protect™ agent were tested in the bioreduction reaction of a symmetrical diketone—benzil (Scheme 1). The purpose of biotransformation catalyzed by Blossom Protect™ was to obtain both chiral 2-hydroxy-1,2-diphenylethanone (benzoin) antipodes in an effective bioconversion reaction characterized by a high catalytic activity of *A. pullulans* and a high enantioselectivity.

### 2.1. Enantioselective Bioreduction Reaction of Benzil to the Corresponding (S)-Benzoin

We first carried out the reduction of benzil (**1**) catalyzed by an antifungal agent containing live *A. pullulans* cells in a phosphate buffer solution at different pH values (pH 5.0 to 8.0). We used glucose as the source of carbon. The reaction was started by adding compound **1**, previously dissolved in ethanol at various concentrations as described in the Experimental section. The mixture was incubated at 30 °C for 24 h. The results are summarized in Table 1.

The effectiveness of bioconversion was strongly affected by both the concentration of substrate and pH of the reaction medium. The highest (up to 92%) ee of (*S*)-benzoin was obtained for 1 × 10^−5^ mol of **1** in a solution at pH 5.5, 6.0, 7.0, 7.2 and 7.5. At a lower pH (5.0–6.0), a gradual decrease in the optical efficiency of the (*S*)-enantiomer was observed with increasing substrate concentration. The phosphate buffer system at pH 5.0, 5.5 and 6.0 showed a slight advantage of the opposite enantiomer with 8–28% ee for (*R*)-benzoin. These results suggest that the activities of different dehydrogenases contained in strains DSM 14940 and DSM 14941 of *A. pullulans* strongly depend on two parameters: pH of the solution and the concentration of the starting reagent. Putatively, in a neutral or slightly alkaline solution (pH 7.2 and 7.5), (*S*)-stereopreference dehydrogenases selectively reduce the symmetrical diaryl diketone, yielding a significant proportion of the (*S*)-isomer irrespective of the substrate concentration. In most cases, the degree of benzil conversion after 24 h of reaction was high (up to 95.5%).

The addition of other sources of carbon, fructose and sucrose, at the pre-incubation stage was also examined. The reaction was carried out at 30 °C. Optimal results were obtained with glucose. It seems that the presence of fructose as a carbon source increases the follow-up reaction, reducing benzoin to the corresponding diol. As a consequence, in most cases, it reduces the enantiomeric purity of hydroxy ketone and its contribution to the post-reaction mixture (Table 2).

Temperature often has a significant impact on bioprocess selectivity, especially in the presence of isolated enzymes, for example hydrolases, or in simple oxidation–reduction models [40,41,42]. Decreasing the temperature usually results in an improvement in enantioselectivity, but may have an adverse effect on the degree of substrate conversion [43,44]. M.C. Fragnelli et al. observed a completely different relationship: reduction of benzil to the corresponding hydroxy ketone, along with a decrease in temperature, was characterized by a slight decrease in the enantiomeric purity of the product with a simultaneous increase in process efficiency. The reduced temperature provides cells with decreased reducing activity towards diol [33].

The bioreduction of **1** was carried out at two additional temperatures (Table 3). Based on previous experiments, glucose was selected as an energy source. It seems that the temperature of 30 °C is optimal, as along with a decrease in temperature to 28 °C, a slight decrease in chemical and optical efficiency and a greater percentage of diol in the post-reaction mixture were observed. A similar observation was made at a higher temperature, and a particular decrease in enantioselectivity was noticed at lower pH (5.5, 6.0).

Attempts have been made to improve the enantioselectivity of biotransformation with the so-called additives. Compounds used as additives can act as hydrogen donors for cofactor regeneration (alcohols), act as inhibitors of enzymes with a particular stereopreference or increase the availability of the substrate for the enzyme (surfactants), and perform chemical modification of the enzyme, increasing its activity (sulfur compounds) [7,45,46,47]. The following potential inhibitors were used in the benzil biotransformation reaction: allyl alcohol, ethyl chloroacetate, cysteine, (9-antryl)glyoxylate (AMA-1), 3-methylbutan-2-one, 4-methylpentan-2-one. The results are shown in Table 4.

Surprisingly, all additives used—to a greater or lesser extent—inhibited the effect of dehydrogenases with (*S*)-stereopreference. The magnitude of these changes depended on the pH of the reaction medium. In the solution at pH 5.0–6.5 in the presence of additives, we observed enrichment of the mixture with the (*R*)-isomer. The highest excess of this isomer, regardless of the pH of the solution, was observed in the biotransformation reaction with the addition of ethyl chloroacetate. In a system with a phosphate buffer at pH 5.5, the optical purity (ee) of the product was 56%. Interestingly, in this solution, the bioreduction reaction proceeded with approximately 50% ee in the presence of each additive, indicating the phosphate buffer solution at pH 5.5 as optimal for the conversion of benzil to (*R*)-benzoin.

Another parameter examined in the benzil bioreduction reaction was the effect of organic solvents on the efficiency and selectivity of the bioprocess. Biocatalysis in a two-phase system is often more effective than in water or polar organic solvents, especially for hydrolases. Enzyme selectivity is conditioned by conformational rigidity, which increases in a more hydrophobic medium. A hydrophobic solvent decreases biocatalyst lability, which prevents binding of a structurally mismatched substrate to the active site of an enzyme [48,49,50]. Water/organic solvent two-liquid-phase systems were successfully used by M.C. Fragnelli et al. and S. Oda et al. in the synthesis of enantiomerically pure (*S*)-benzoin [33,34]. The enantioselectivity of the reaction was increased in hydrophobic solvents such as hexane, heptane, and isooctane. The presence of a cosolvent promoted the deracemization reaction without the simultaneous occurrence of reduction activities towards benzoin [33]. We bioreduced benzil with added cosolvents using hexane, cyclohexane, *tert*-butyl methyl ether (TBME), tetrahydrofuran (THF), acetonitrile (AcCN), and ionic liquids (Bmim)(BF_4_) and (Bmim)(PF_6_) (Table 5). No increase in the optical efficiency of the (*S*)-isomer was observed; however, after 24 h in a solution of phosphate buffer mixed with organic solvents at a 10:1 ratio (*v*/*v*), the presence of TBME, AcCN, and THF promoted the growth of (*R*)-benzoin in the mixture. The highest excess of the (*R*)-enantiomer was observed in the buffer: THF and buffer: AcCN mixtures at pH 5.5 and 6.0, respectively.

For the evaluation of the enantioselective properties of the microorganism in benzil reduction, a time profile was established by determining the composition of the reaction mixture over time. The reaction was stopped after 2 and 4 h. Optimal conditions were selected, incubation at 30 °C with glucose as energy source and 1 × 10^−5^ mol of substrate. The results are shown in Table 6. Both in the second and fourth hour, the reaction was practically non-selective. After two hours, the highest ee of (*S*)-benzoin was obtained in solutions at pH 7.0 and 7.5: 61% and 66%, respectively. However, after 4 h, in each case a slight increase in the (*R*)-isomer content in the mixture was observed, similarly as after 2 h in a solution with pH 5.0–6.0. This result is in apparent contradiction to the previous hypothesis that for 1 × 10^−5^ mol of **1**, dehydrogenase with (*S*)-stereopreference selectively reduces the symmetrical diaryl diketone. It seems that only stereoinversion (deracemization) of the stereogenic benzoin atom can explain the situation. Stereoinversion of secondary alcohols can occur by concurrent tandem biocatalytic oxidation and reduction. If one of the enantiomers is selectively oxidized to carbonyl and then there is a selective reduction to the opposite enantiomer, enantiomeric enrichment of the mixture with the specified enantiomer can be observed. For this reason, a significant contribution of the (*S*)-isomer is observed after 24 h.

This is confirmed by data in the literature, which show that during the reduction of benzil using microbiological methods, selective oxidation of (*R*)-benzoin to benzil occurs. Increased oxidative activity was observed by M.C. Fragnelli et al. in a two-liquid-phase system [33] and by A. S. Demir et al. in a phosphate buffer at different pH values [31]. The process of stereoinversion might be caused by the activity of different dehydrogenases: one of them selectively oxidizes (*R*)-benzoin to diketone and the other—(*S*)-selective dehydrogenase with a high affinity towards benzil—reduces it to the (*S*)-isomer. In order to determine how stereoinversion affects the optical purity of the product, we biotransformed *rac*-benzoin over time (Table 7). After 2 h, there was practically no reduction to diol, only deracemization which led to a significant percentage of the (*S*)-enantiomer (up to 95% ee), due to the selective oxidation of the isomer of the opposite configuration. Kinetic resolution of the racemate through enantioselective oxidation was observed. In the fourth hour, the (*S*)-enantiomer contribution was modulated by three competitive reactions: oxidation of benzoin to benzil, reduction of benzil to benzoin, and bioconversion of benzoin to hydrobenzoin. An increased percentage of benzil and (*R*,*R*)-hydrobenzoin (pH 5.0, 6.0–7.0, 7.5, and 8.0) or (*S*,*S*)-hydrobenzoin (pH 5.5 and 7) was observed. However, after 24 h, the situation was further complicated due to the presence of an additional oxidation reaction of the previously obtained hydrobenzoin to benzoin. The optical purity of benzoin was a result of all these competitive reactions. The highest ee of (*S*)-enantiomer was obtained in a solution with pH 6.5 (84%).

### 2.2. Enantioselective Bioreduction Reaction of Benzil to the Corresponding (R)-Benzoin

The increase in substrate concentration contributed to the reduced proportion of (*S*)-benzoin in the mixture after 24 h of reaction, especially for lower pH values (Table 1). In order to obtain an excess of the *R*-configuration enantiomer, we decided to bioreduce the prochiral diketone in a solution with pH 5.0 and 5.5 for higher reagent concentrations. The results are shown in Table 8.

The change in stereobias with increasing concentration of substrate could be explained taking into account the possible presence of more than one alcohol dehydrogenase with different enantioselectivity inside the cell. Oxidoreductases, contained in the microorganism, act competitively to each other by transferring the hydride (pro-*S* or pro-*R*) from the nicotinamide adenine dinucleotide (NAD(P)H) cofactor to one of the sides of the prochiral carbonyl group (face *si* or *re*). The final ee of the product results from the reduction reactions occurring at different rates, carried out by a set of oxidoreductases, and the hydroacetone deracemization reaction. We observed that with the increase of reagent concentration, the reduction reaction proceeded more slowly, so that after the prolonged reaction time, another dehydrogenase could start acting. Under favorable conditions, with a correspondingly higher substrate concentration and prolonged reaction time, the (*S*)-isomer of benzil can be deracemized. The diketone can then be converted by (*R*)-dehydrogenase to the corresponding (*R*)-benzoin, despite the (*S*)-selective dehydrogenase showing a higher affinity for benzil. The effectiveness of bioconversion is usually strongly affected by substrate concentrations. A decrease in the catalytic activity of the microorganism is observed to increase as the concentration of the reagent increases. It can be presumed that too high a concentration of the substrate may be inhibitory to dehydrogenases, especially those with (*S*)-stereopreference, resulting in a decrease in their chemical efficiency and an increased contribution of the enantiomer of the *R* configuration. Finally, after 5 days, up to 79% ee was obtained for (*R*)-benzoin in a solution at pH 5.5. However, the highest ee of 93% was obtained after 7 days in a solution at pH 5.0 for 1 × 10^−4^ mol of **1**.

For 5 × 10^−5^ of **1**, benzil reduction was carried out in the presence of potential inhibitors (Table 9). Compared with the results presented in Table 4, a significant effect of reagent concentration on the enantioselectivity of the bioprocess was observed. In each case, the reaction mixture was enriched by the (*R*)-isomer, although with a fairly low degree of conversion.

In the most promising cases with ee above 60%, the diketone bioreduction reaction was performed using selected (*S*)-dehydrogenase inhibitors under the same conditions and with the reaction time prolonged to 5 days. Results are shown in Table 10.

As a result, in solutions with a pH value of 7.0 to 7.5, selectivity and biotransformation efficiency were improved in the presence of selected additives. Optimal results were obtained in a phosphate buffer solution of pH 7.0, using 4-methylpentan-2-one as selective inhibitor. Ee reached 91%.

The effect of additives on the selectivity of *rac*-benzoin bioconversion was subsequently determined. As the highest enantiomeric excess, in most cases (except cysteine and ethyl chloroacetate), was obtained in a solution with a pH value of 7.5, the reaction under these conditions was carried out for both amount of *rac*-**2**: 1 × 10^−5^ and 5 × 10^−5^ mol (Table 11).

Surprisingly, the bioconversion reaction of *rac*-benzoin at a reagent amount of 1 × 10^−5^ mol was not selective. A significant proportion of diketone in the mixture was found, which suggests promoting oxidation rather than reduction. On the other hand, for a higher concentration of the reagent, besides the non-selective deracemization reaction, an increase in the bioreduction reaction rate was observed. In the presence of 3-methylbutan-2-one and 4-methylpentan-2-one, diol with the (*R*,*R*) configuration was obtained as the main product, pointing out the occurrence of the reduction activity of dehydrogenases. The (*R*,*R*): *meso* ratio was 2.5:1 and 2.3:1 for 3-methylbutan-2-one and 4-methylpentan-2-one, respectively. The (*S*,*S*)-isomer was detected using reaction with cysteine ((*S*,*S*): *meso* 1.7:1). In other cases, the predominant isomer obtained was the *meso* compound. The highest composition of this isomer was accumulated in the reaction medium using ethyl chloroacetate as additive ((*S*,*S*): *meso* 1:6). Based on the results obtained, it can be assumed that by performing a more detailed optimization of the reaction conditions, it will be possible to obtain a diol with the desired configuration.

## 3. Experimental Section

### 3.1. Materials and Methods

#### 3.1.1. General Informations

The enantiomeric excess of the chiral products was determined by a chiral stationary phase of high-performance liquid chromatography (HPLC). HPLC analyses were performed on a Shimadzu SCL-10A *VP (*Kyoto, Japan).

Compound **1,** (*S*)-**2** and (*R*)-**2** was separated on a column Lux^®^ 5µ Cellulose-1, LC Column 250 × 4.6 mm, Phenomenex (Warsaw, Poland). The mobile phase was *n*-hexane and propan-2-ol (90:10 *v*/*v*), at the flow rate of 0.6 mL per min. The retention times of **1,** (*S*)-**2** and (*R*)-**2** were 9.2 min, 20.1 min and 30.0 min, respectively.

Compound (*R*,*R*)-**3**, (*S*,*S*)-**3** and (*S*,*R*)-**3** were separated on a column Lux^®^ 5µ Cellulose-3, LC Column 250 × 4.6 mm, Phenomenex (Poland). The mobile phase was *n*-hexane and propan-2-ol (90:10 *v*/*v*), at the flow rate of 0.6 mL per min. The retention times of (*R*,*R*)-**3**, (*S*,*S*)-**3** and *meso*-**3** were 21.6 min, 28.4 min and 26.2 min, respectively.

The samples were incubated in an orbital shaker (VORTEMP 1550 S2050; Equimed, Cracow, Poland).

The absolute configurations of (*S*)-**2,** (*R*)-**2**, (*R*,*R*)-**3**, (*S*,*S*)-**3** and *meso*-**3** were assigned based on the configuration of standards purchased from Merck (Darmstadt, Germany).

#### 3.1.2. Reagents and Solvents

The chemical substances of analytical grade were commercially available and they included: sucrose, glucose, fructose, ethyl acetate, ethanol, *n*-hexane, cyclohexane, *tert*-butyl methyl ether (TBME), acetonitrile (AcCN), tetrahydrofuran (THF), allyl alcohol, ethyl chloroacetate, MgSO_4_, Na_2_HPO_4_, NaH_2_PO_4_, *n*-hexane for HPLC, propan-2-ol for HPLC from POCH (Gliwice, Poland), benzil, *rac*-benzoin, (*S*)-benzoin, (*R*)-benzoin, (*R*,*R*)-hydrobenzoin, *meso*-hydrobenzoin, cysteine, 3-methylbutan-2-one, 4-methylpentan-2-one, 1-butyl-3-methylimidazolium tetrafluoroborate ((Bmim)(BF_4_) from Merck (Darmstadt, Germany). 1-butyl-3-methylimidazolium hexafluorophosphate ((Bmim)(PF_6_), Fluka, Switzerland). Blossom Protect™ from Koppert Biological Systems (Wien, Austria) contains germinating fungal *Aureobasidium pullulans* strains DSM 14940 and 14941 (500 g per 1 kg of the preparation).

### 3.2. Bioreduction of **1** by Blossom Protect™ Contains Aureobasidium pullulans Strains DSM 14940 and 14941

For a typical experiment, to a suspension of Blossom Protect™ (1 g) in 7.5 mL of potassium phosphate buffer was added 1 × 10^−4^ mol glucose (Table 1, Table 3, Table 4, Table 5, Table 6, Table 8, Table 9 and Table 10) or fructose, sucrose (Table 2) and the resulting suspension was incubated in an orbital shaker (350 rpm) for 30 min at 30 °C.

#### 3.2.1. I

After pre-incubation, benzil **1** was added in the following amounts: 5 × 10^−6^, 1 × 10^−5^, 2 × 10^−5^, 3 × 10^−5^, 4 × 10^−5^, 5 × 10^−5^ moles (Table 1); 6 × 10^−5^, 7 × 10^−5^, 8 × 10^−5^, 9 × 10^−5^, 1 × 10^−5^ moles (Table 8). Substrate was dissolved in 0.5 mL of ethanol and added to the suspension of biocatalyst in phosphate buffer at the following pH values: 5.0, 5.5, 6.0, 6.5, 7.0, 7.2, 7.5, 8.0 (Table 1) or 5.0 and 5.5 (Table 8). Next Falcon tubes were stirred at same temperature at different intervals: 24 h (Table 1/Table 8); 48 h, 120 h, 168 h (Table 8).

#### 3.2.2. II

After pre-incubation, 1 × 10^−5^ mole of benzil dissolved in 0.5 mL of ethanol were added to the suspension of biocatalyst in phosphate buffer at the following pH values: 5.0, 5.5, 6.0, 6.5, 7.0, 7.2, 7.5, 8.0. Next Falcon tubes were stirred 24 h at 28 °C, 32 °C (Table 3) or 0.5 h, 2 h and 4 h (Table 6) 24 h (Table 2) at 30 °C.

#### 3.2.3. III

After pre-incubation, 1 × 10^−4^ mole of benzil dissolved in 0.5 mL of ethanol was added to biphasic system composed by phosphate buffer at the following pH values: 5.0, 5.5, 6.0, 6.5, 7.0, 7.2, 7.5, 8.0 and organic solvent: *n*-hexane, cyclohexane, TBME, THF, AcCN, (Bmim)(PF_6_), (Bmim)(BF_4_) (buffer:organic solvent 10:1 *v*/*v*). Next Falcon tubes were stirred 24 h at 30 °C (Table 5).

#### 3.2.4. IV

After pre-incubation, 1 × 10^−5^ (Table 4) or 1 × 10^−5^ (Table 9 and Table 10) mole of benzil dissolved in 0.5 mL of ethanol, additives: allyl alcohol/ethyl (9-antryl)glyoxylate (AMA-1)/cysteine/3-methyl-2-butanone/4-methyl-2-pentanone/ethyl chloroacetate (1.25 × 10^−5^) were added to the suspension of biocatalyst in phosphate buffer at the following pH values: 5.0, 5.5, 6.0, 6.5, 7.0, 7.2, 7.5, 8.0. Next Falcon tubes were stirred 24 h at 30 °C (Table 4, Table 9 and Table 10).

The reaction progress was monitored by TLC (the solvent system used was *n*-hexane: ethyl acetate 4:1 *v*/*v*). After the reaction was completed, microorganism was filtered off the supernatant, washed with distilled water. The solid residue was washed with ethyl acetate and the combined aqueous phases were extracted with ethyl acetate (3 × 20 mL). The collected organic layer was dried over anhydrous MgSO_4_ and the solvent was evaporated in a vacuum. The conversion degrees of the substrates and enantiomeric ratios of the products were determined on HPLC system using a chiral column.

### 3.3. Biotransformation of Rac-**2** by Blossom Protect™ Contains Aureobasidium pullulans Strains DSM 14940 and 14941

Biotransformation reaction was preceded by half an hour pre-incubation on a rotary shaker at 350 revolutions per minute (rpm) at 30 °C. The composition of the pre-incubation mixture was as follows: 1 × 10^−4^ mol glucose, 1 g Blossom Protect™, 7.5 mL phosphate buffer (pH 5.0, 5.5, 6.0, 6.5, 7.0, 7.2, 7.5, 8.0). After pre-incubation, 1 × 10^−5^ (Table 7/Table 11) or 5 × 10^−5^ (Table 11) moles of *rac*-benzoin dissolved in 0.5 mL of ethanol were added to the mixture and optionally additives (Table 11). After completion of the reaction, the mixture was filtered. Then, the filtrate and the solid phase were extracted four times with ethyl acetate (25 mL) and the organic layer was evaporated. The conversion degrees of the substrates and enantiomeric ratios of the products were determined on HPLC system using a chiral column.

### 3.4. Blossom Protect-Mediated Synthesis of (S)-**2**/(R)-**2**—Semi-Preparative-Scale

Compounds (*S*)- and (*R*)-**2** were obtained on a semi-preparative scale using previously optimized reaction conditions.

#### 3.4.1. A. Synthesis of (*S*)-**2**

A suspension of Blossom Protect^TM^ agent (40 g) and glucose (1.44 g) in phosphate buffer (pH 7.2, 300 mL) was maintained for 30 min at 30 °C with stirring (350 rpm). After this time, to the fermenting mixture, a solution of **1** (0.4 mmol; 95.2 mg) in EtOH (10 mL) was added and the resulting mixture was vigorously stirred at 30 °C using laboratory orbital shaker (350 rpm) under anaerobic conditions for 24 h. The rest of the reaction work-up were performed in analogy to the previously described analytical-scale reactions with obvious preservation of the appropriate the reaction parameters. The crude products have been purified by column chromatography using mixture of *n*-hexane/AcOEt (7:3, *v*/*v*) as an eluent to afford the respective (*S*)-**2** (49.5 mg, 0.24 mmol, 85% conv., 52% yield; 90% ee).

#### 3.4.2. B. Synthesis of (*R*)-**2**

A suspension of Blossom Protect^TM^ agent (40 g) and glucose (1.44 g) in phosphate buffer (pH 7.0, 300 mL) was maintained for 30 min at 30 °C with stirring (350 rpm). After pre-incubation, ethanolic solution of **1** (EtOH 10 mL) was added in the amount of 0.4 mmol (95.2 mg) and 4-methylpentan-2-one (63 µL). The resulting mixture was vigorously stirred at 30 °C using laboratory orbital shaker (350 rpm) under anaerobic conditions. After 2 days biomass was removed by filtration. The bio-reduction product ((*R*)-**2**) was eluted from the aqueous phase and the recovered biomass with ethyl acetate. The rest of the reaction work-up were performed in analogy to the previously described analytical-scale reactions with obvious preservation of the appropriate the reaction parameters. The crude products have been purified by column chromatography using mixture of *n*-hexane/AcOEt (7:3, *v*/*v*) as an eluent to afford the respective (*R*)-**2** (34.3 mg, 0.24 mmol, 68% conv., 36% yield; 90% ee).

## 4. Conclusions

In conclusion, this work shows that slight modifications of the reaction conditions grant the opportunity to obtain 2-hydroxy-1,2-diphenylethanone with defined chirality on an asymmetric carbon atom. In the enzymatic bioreduction applying the catalytic properties of the antifungal agent, both enantiomers can be obtained with good optical efficiency.

The product with *S* configuration ((*S*)-**2**) with the highest enantiomeric excess (approximately 90%) was received by reducing **1** in a phosphate buffer solution at pH 5.5–7.5 for 24 h at 30 °C with 1 × 10^−5^ mol of substrate, as well as after a two-hour incubation of 1 × 10^−5^ mol *rac*-**2** at 30 °C in an aqueous medium with a pH value of 6.5–7.5.

Benzil was effectively reduced to (*R*)-**2** with good stereoselectivity up to 93% optical purity in a biotransformation of 10 × 10^−5^ moles of **1** conducted for 168 h in a solution of slightly acidic pH 5.0 at 30 °C. (*R*)-isomer with high enantiomeric excess was also obtained in phosphate buffer pH 7.0–7.5 with the use of additives (allyl alcohol, AMA-1, 3-methylbutan-2-one, 4-methylpentan-2-one) for 5 × 10^−5^ moles of substrate. After 120 h of incubation with 4-methylpentan-2-one, Blossom Protect^TM^-mediated biotransformation of **1** was carried out with good stereoselectivity, allowing for obtaining an enantiomeric excess of up to 91%.

The performed studies made it possible to formulate the conclusion that the synthesis of (*S*)-benzoin is possible thanks to the enzymatic desymmetrization of benzil and deracemization of benzoin if the reaction is carried out in mild reaction conditions: neutral environment at 30 °C for 24 h. Reagent concentration and the additives (potential inhibitors, co-solvents) affect change of selective properties of the microorganism. Along with the increase in substrate concentration, decreased pH value, prolonged reaction time and addition of substances “poisoning” the protein catalyst with defined chirality, preferentially an enantiomer with the opposite configuration, was obtained. The substrate, like the additive, acts as an inhibitor, selectively inhibiting the action of *S*-dehydrogenases.

## Figures and Tables

**Table 1:** Microbial Bioreduction of Benzil (**1**) to (*S*)-2-Hydroxy-1,2-Diphenylethanone (**2**) in Phosphate Buffer at Different pH Values and Various Concentrations of **1**.

pH	Conv. (%)/ee (%)	Amount of Substrate × 10^−5^ (mol)					
		5 × 10^−1^	1	2	3	4	5
**5.0**	Conv. (%)/	74.4	79.8	75.5	72.7	52.1	76.9
	ee (%)	74 (*S*)	86 (*S*)	58 (*S*)	21 (*S*)	11 (*R*)	21 (*R*)
**5.5**	Conv. (%)/	81.3	89.3	87.4	74.8	66.7	63.9
	ee (%)	83 (*S*)	91 (*S*)	83 (*S*)	32 (*S*)	8 (*R*)	28 (*R*)
**6.0**	Conv. (%)/	83.7	86.4	81.1	80.9	66.1	79.1
	ee (%)	80 (*S*)	90 (*S*)	87 (*S*)	81 (*S*)	6 (*S*)	8 (*R*)
**6.5**	Conv. (%)/	80.8	83.2	82.2	82.4	80.6	93.3
	ee (%)	76 (*S*)	89 (*S*)	82 (*S*)	74 (*S*)	67 (*S*)	66 (*S*)
**7.0**	Conv. (%)/	86.7	88.1	86.4	84.8	89.8	94.4
	ee (%)	80 (*S*)	90 (*S*)	87 (*S*)	84 (*S*)	82 (*S*)	77 (*S*)
**7.2**	Conv. (%)/	84.7	87.6	86.3	93.5	90.2	91.8
	ee (%)	75 (S)	92 (*S*)	87 (*S*)	88 (*S*)	89 (S)	87 (S)
**7.5**	Conv. (%)/	81.0	75.3	87.9	91.6	89.3	93.8
	ee (%)	73 (*S*)	91 (*S*)	92 (*S*)	86 (*S*)	84 (*S*)	82 (*S*)
**8.0**	Conv. (%)/	77.0	89.1	90.2	95.5	89.9	89.4
	ee (%)	67 (*S*)	77 (*S*)	70 (*S*)	67 (*S*)	62 (*S*)	60 (*S*)

**Table 2:** The Efficiency and Enantioselectivity of the Bioconversion of **1** by *A. pullulans* Strain DSM 14940 and DSM 14941 with Fructose and Sucrose as Energy Source.

Conditions 30 °C; 1 × 10^−5^ mola of 1	(*S*)-2	pH							
		5.0	5.5	6.0	6.5	7.0	7.2	7.5	8.0
**glucose**	Conv. (%)/ee (%)	79.8/86	89.3/91	86.4/90	83.2/89	88.1/90	87.6/92	75.3/91	89.1/77
**fructose**	Conv. (%)/ee (%)	82.7/89	76.9/73	76.5/81	78.7/76	85.8/72	63.4/72	86.1/69	86.5/60
**sucrose**	Conv. (%)/ee (%)	78.2/87	82.5/88	80.7/88	89.2/86	88.8/82	69.5/75	89.5/82	87.6/71

**Table 3:** The Efficiency and Enantioselectivity of the Bioconversion of **1** at 28 °C, 30 °C and 32 °C by *A. pullulans* Strain DSM 14940 and DSM 14941.

Conditions Glucose; 1 × 10^−5^ mola of 1	(*S*)-2	pH							
		5.0	5.5	6.0	6.5	7.0	7.2	7.5	8.0
**28 °C**	Conv. (%)/ee (%)	80.0/86	81.1/87	77.9/87	80.8/85	83.0/85	84.0/87	80.7/87	81.5/81
**30 °C**	Conv. (%)/ee (%)	79.8/86	89.3/91	86.4/90	78.7/89	88.1/90	87.6/92	87.9/92	89.1/77
**32 °C**	Conv. (%)/ee (%)	78.5/67	70.0/49	63.0/41	78.5/76	78.2/72	73.6/86	86.0/73	85.3/62

**Table 4:** Microbial Bioreduction of **1** in Phosphate Buffer at Different pH Values with Additives.

pH	Conv. (%)/ee (%)	Additives					
		Allyl Alcohol	Ethyl Chloroacetate	Cysteine	AMA-1	3-Methylbutan-2-one	4-Methylpentan-2-one
**5.0**	Conv. (%)	24.0	35.5	58.3	22.3	62.2	54.0
	ee (%)	44 (*R*)	42 (*R*)	43 (*R*)	41 (*R*)	40 (*R*)	34 (*R*)
**5.5**	Conv. (%)	10.5	9.2	41.9	27.3	33.6	43.9
	ee (%)	48 *(R*)	56 *(R*)	51 (*R*)	51 (*R*)	50 (*R*)	51 (*R*)
**6.0**	Conv. (%)	23.9	20.8	29.3	42.0	50.2	53.8
	ee (%)	44 (*R*)	48 (*R*)	41 (*R*)	46 (*R*)	48 (*R*)	16 (*R*)
**6.5**	Conv. (%)	35.9	25.2	52.3	59.1	50.4	30.5
	ee (%)	46 (*R*)	55 (*R*)	18 (*R*)	9 (*R*)	43 (*R*)	38 (*R*)
**7.0**	Conv. (%)	45.7	16.5	85.7	79.6	48.2	44.9
	ee (%)	24 (*R*)	54 (*R*)	88 (*S*)	75 (*S*)	46 (*R*)	33 (*R*)
**7.2**	Conv. (%)	42.4	19.6	86.0	70.2	62.7	49.6
	ee (%)	46 (*R*)	51 *(R*)	73 (*S*)	41 (*S*)	17 (*R*)	40 (*R*)
**7.5**	Conv. (%)	56.5	15.0	73.4	79.3	48.3	64.7
	ee (%)	26 (*R*)	52 (*R*)	83 (*S*)	89 (*S*)	45 (*R*)	31 (*R*)
**8.0**	Conv. (%)	56.8	41.2	84.9	73.3	53.8	67.5
	ee (%)	4 (*R*)	48 (*R*)	82 (*S*)	10 (*R*)	22 (*R*)	1 (*R*)

**Table 5:** Bioreduction of **1** Catalyzed by *A. pullulans* Strain DSM 14940 and DSM 14941 in a Two Liquid-Phase Systems.

pH	Conv. (%)/ee (%)	Organic Solvent: Phosphate Buffer 1:10 *v*/*v*						
		Hexane	Cyclohexane	TBME	(Bmim)(PF_6_)	(Bmim)(BF_4_)	THF	AcCN
**5.0**	Conv. (%)	59.9	67.8	12.9	34.3	74.9	11.4	14.9
	ee (%)	81 (*S*)	74 (*S*)	43 (*R*)	6 (*R*)	13 (*R*)	52 (*R*)	48 (*R*)
**5.5**	Conv. (%)	63.7	64.8	8.3	70.4	47.9	11.0	12.2
	ee (%)	83 (*S*)	67 (*S*)	45 (*R*)	9 (*R*)	45 (*R*)	56 (*R*)	48 (*R*)
**6.0**	Conv. (%)	69.3	59.4	9.6	22.4	76.8	17.3	23.4
	ee (%)	86 (*S*)	73 (*S*)	46 (R)	3 (*R*)	26 (*S*)	42 (*R*)	55 *(R*)
**6.5**	Conv. (%)	64.7	69.4	19.9	37.9	73.7	11.7	27.7
	ee (%)	84 (*S*)	82 (*S*)	42 (*R*)	29 (*S*)	80 (*S*)	40 (*R*)	52 (*R*)
**7.0**	Conv. (%)	70.6	83.4	25.4	36.1	77.6	16.4	29.0
	ee (%)	81 (*S*)	75 (*S*)	40 (*R*)	40 (*S*)	81 (*S*)	40 (*R*)	52 (*R*)
**7.2**	Conv. (%)	75.5	92.5	31.9	46.4	78.6	11.4	18.5
	ee (%)	86 (*S*)	77 (*S*)	41 (*R*)	41 (*S*)	41 (*S*)	25 (*R*)	35 (*R*)
**7.5**	Conv. (%)	66.1	91.2	33.1	51.2	76.7	17.2	17.1
	ee (%)	81 (*S*)	69 (*S*)	33 (*R*)	42 (*S*)	55 (*S*)	35 (*R*)	26 (*R*)
**8.0**	Conv. (%)	71.3	89.0	39.7	47.6	41.9	14.3	11.5
	ee (%)	44 (*S*)	36 (*S*)	35 (*R*)	21 (S)	26 (*R*)	32 (*R*)	7 (*R*)

**Table 6:** Time Profile of Bioreduction of **1** Catalyzed by *A. pullulans* Strain DSM 14940 and DSM 14941 in Phosphate Buffer at Different pH Values.

Conditions	pH	Time (h)	Conv. (%)	Conv. (%)		ee (%)
			1	2		
				(*S*)	(*R*)	
**phosphate buffer; 30 °C; 1 × 10^−5^ mol of 1**	5.0	2	28.0	27.0	45.0	25 (*R*)
	5.5	2	43.7	18.9	37.4	33 (*R*)
	6.0	2	39.6	24.5	35.9	19 (*R*)
	6.5	2	23.4	42.1	34.4	10 (*S*)
	7.0	2	13.3	72.1	14.5	66 (*S*)
	7.2	2	19.3	54.7	25.9	36 (*S*)
	7.5	2	10.9	71.5	17.5	61 (*S*)
	8.0	2	38.4	23.2	38.3	25 (*S*)
	5.0	4	79.7	5.6	14.7	45 (*R*)
	5.5	4	68.3	10.1	20.2	33 (*R*)
	6.0	4	47.9	21.6	29.3	15 (*R*)
	6.5	4	46.6	23.8	28.2	8 (*R*)
	7.0	4	34.2	26.4	36.3	11 (*R*)
	7.2	4	50.6	19.3	30.2	22 (*R*)
	7.5	4	44.3	27.5	28.2	1 (*R*)
	8.0	4	65.9	14.2	19.3	15 (*R*)

**Table 7:** Deracemization of *Rac*-**2** Catalyzed by *A. pullulans* Strain DSM 14940 and DSM 14941 in Phosphate Buffer at Different pH Values.

Conditions	pH	Time (h)	Yield (%)	Yield (%)		ee (%)	Yield (%)		
			1	2			Hydrobenzoin		
				(*S*)	(*R*)		(*R*,*R*)	(*S*,*S*)	*Meso*
**phosphate buffer; 30 °C; 1 × 10^−5^ mola of *rac*-2**	5.0	2	18.5	87.0	8.6	82 (*S*)	7.4	0.4	1.4
	5.5	2	13.6	68.5	11.4	71 (*S*)	4.5	0.4	1.5
	6.0	2	13.3	80.6	6.1	86 (*S*)	-	-	-
	6.5	2	11.5	78.0	2.2	94 (*S*)	6.7	0.7	0.8
	7.0	2	12.6	85.7	2.2	95 (*S*)	2.8	1.5	1.5
	7.2	2	12.9	74.3	2.2	94 (*S*)	8.1	0.5	2.0
	7.5	2	11.7	70.8	3.6	90 (*S*)	10.3	0.5	3.2
	8.0	2	42.0	44.1	6.2	75 (*S*)	0.5	0.2	2.6
	5.0	4	43.2	15.8	3.0	68 (*S*)	13.1	34.9	-
	5.5	4	49.7	8.4	4.0	35 (*S*)	26.1	11.7	-
	6.0	4	24.1	4.1	1.0	61 (*S*)	14.3	56.5	-
	6.5	4	29.8	4.8	1.8	45 (*S*)	17.2	46.4	-
	7.0	4	29.1	4.4	2.0	38 (*S*)	22.4	42.1	-
	7.2	4	52.7	7.6	3.2	41 (*S*)	26.0	10.4	-
	7.5	4	44.5	16.4	7.1	40 (*S*)	9.5	11.6	10.9
	8.0	4	51.9	18.1	10.2	28 (*S*)	2.6	5.9	11.2
	5.0	24	31.3	49.3	7.9	72 (*S*)	4.0	1.4	6.1
	5.5	24	37.4	39.7	8.4	65 (*S*)	8.1	0.7	5.6
	6.0	24	74.6	9.3	5.5	26 (*S*)	9.9	0.4	0.3
	6.5	24	53.1	33.1	2.9	84 (*S*)	4.8	0.3	5.8
	7.0	24	48.4	26.3	6.5	60 (*S*)	4.3	0.1	14.3
	7.2	24	48.7	24.7	8.1	51 (*S*)	4.3	0.2	13.9
	7.5	24	52.2	19.6	8.4	40 (*S*)	5.4	0.2	13.1
	8.0	24	55.4	19.4	10.9	28 (*S*)	2.2	0.2	11.9

**Table 8:** Microbial Bioreduction of **1** to (*R*)-2-Hydroxy-1,2-Diphenylethanone (**2**) in Phosphate Buffer at Different pH Values and Various Concentrations of **1**.

Amount of Substrate× 10^−5^ (mol)	pH			
	5.0		5.5	
	Conv. (%)	ee (%) of (*R*)-2	Conv. (%)	ee (%) of (*R*)-2
**6**	57.7 ^a^/67.6 ^d^	21 ^a^/13 ^d^	69.0 ^a^/83.5 ^d^	23 ^a^/4 ^d^
**7**	37.8 ^a^/90.7 ^c^/69.1 ^d^	35 ^a^/73 ^c^/61 ^d^	51.8 ^a^/84.0 ^c^/85.8 ^d^	36 ^a^/79 ^c^/59 ^d^
8	37.8 ^a^/78.6 ^c^/70.6 ^d^	29 ^a^/66 ^c^/17 ^d^	24.9 ^a^/80.8 ^c^/47.2 ^d^	35 ^a^/65 ^c^/76 ^d^
**9**	24.5^a^ /18.3 ^b^/48.0 ^c^/53.8 ^d^	38 ^a^/33 ^b^/71 ^c^/66 ^d^	22.0 ^a^/41.3 ^b^/69.4 ^c^/57.7 ^d^	36 ^a^/8 ^b^/71 ^c^/74 ^d^
**10**	22.6 ^a^/47.0 ^c^/15.6 ^d^	30 ^a^/68 ^c^/93 ^d^	25.1 ^a^/26.9 ^b^/62.2 ^c^/36.9 ^d^	37 ^a^/47 ^b^/75 ^c^/63 ^d^

^a^ 24 h; ^b^ 48 h; ^c^ 120 h; ^d^ 168 h.

**Table 9:** Microbial Bioreduction of **1** Catalyzed by *A. pullulans* Strain DSM 14940 and DSM 14941 in Phosphate Buffer at Different pH Values with Additives.

pH	Conv. (%)/ee (%)	Additives					
		Allyl Alcohol	Ethyl Chloroacetate	Cysteine	AMA-1	3-Methylbutan-2-one	4-Methylpentan-2-one
**5.0**	Conv. (%)	0.9	2.8	8.4	2.8	0.7	0.6
	ee (%)	78 (*R*)	22 (*R*)	57 (*R*)	59 (*R*)	59 (*R*)	54 (*R*)
**5.5**	Conv. (%)	15.3	25.9	1.8	2.0	13.6	5.0
	ee (%)	39 (*R*)	52 (*R*)	56 (*R*)	60 (*R*)	41 (*R*)	42 (*R*)
**6.0**	Conv. (%)	4.9	30.4	13.5	31.2	12.4	30.8
	ee (%)	75 (*R*)	54 (*R*)	51 (*R*)	58 (*R*)	46 (*R*)	51 (*R*)
**6.5**	Conv. (%)	10.0	18.9	54.5	39.6	18.7	46.5
	ee (%)	54 (*R*)	53 (*R*)	29 (*R*)	53 (*R*)	54 (*R*)	43 (*R*)
**7.0**	Conv. (%)	2.7	0.2	9.5	18.9	4.5	5.9
	ee (%)	56 (*R*)	45 (*R*)	57 (*R*)	59 (*R*)	63 (*R*)	60 (*R*)
**7.2**	Conv. (%)	4.9	1.9	22.1	6.6	10.2	16.9
	ee (%)	60 (*R*)	46 (*R*)	52 (*R*)	58 (*R*)	55 (*R*)	53 (*R*)
**7.5**	Conv. (%)	26.7	2.3	30.8	18.1	7.9	19.6
	ee (%)	63 (*R*)	29 (*R*)	53 (*R*)	61 (*R*)	65 (*R*)	68 (*R*)
**8.0**	Conv. (%)	32.2	24.4	49.1	30.3	11.1	14.7
	ee (%)	48 (*R*)	64 (*R*)	45 (*R*)	34 (*S*)	57 (*R*)	54 (*R*)

**Table 10:** Bioreduction of **1** Catalyzed by *A. pullulans* Strain DSM 14940 and DSM 14941 in Specific Conditions with Selected Additives.

Conditions	pH	Additives	Conv. (%)	ee (%)
**phosphate buffer; 30** **°** **C; 5 × 10^−5^ mola of 1**	5.0	Allyl alcohol	7.9	32 (*R*)
	6.0	Allyl alcohol	9.7	72 (*R*)
	7.2	Allyl alcohol	60.9	83 (*R*)
	7.5	Allyl alcohol	45.9	88 (*R*)
	7.0	4-Methylpentan-2-one	71.1	91 (*R*)
	7.0	3-Methylbutan-2-one	88.5	89 (*R*)
	7.5	4-Methylpentan-2-one	82.9	87 (*R*)
	7.5	3-Methylbutan-2-one	48.9	84 (*R*)
	5.5	AMA-1	3.8	37 (*R*)
	7.5	AMA-1	43.1	83 (*R*)
	8.0	Ethyl chloroacetate	50.8	41 (*R*)

**Table 11:** Deracemization of *Rac-*Benzoin (**2**) and Bioreduction Catalyzed by *A. pullulans* Strain DSM 14940 and DSM 14941 in Phosphate Buffer with Additives.

Conditions	pH	Additives	Conv. (%)	Conv. (%)		ee (%) of 2	Conv. (%)		
			1	2			3		
				(*S*)	(*R*)		(*R*,*R*)	(*S*,*S*)	*meso*
**Phosphate buffer;30** **°** **C; 1 × 10^−5^ mola of 1; 24 h**	7.5	Allyl alcohol	41.9	24.6	30.2	10 (*R*)	0.8	2.5	-
	7.5	AMA-1	38.1	29.8	27.9	3 (*S*)	3.4	0.7	-
	7.0	Cysteine	51.1	26.6	20.5	13 (*S*)	1.4	0.4	-
	7.5	3-Methylbutan-2-one	35.1	33.1	25.9	12 (*S*)	1.6	4.2	-
	7.5	4-Methylpentan-2-one	54.4	22.8	22.9	*rac*	-	-	-
	8.0	Ethyl chloroacetate	65.3	20.4	10.6	32 (*S*)	1.9	0.9	0.9
**Phosphate buff;30 °C; 5 × 10^−5^ mola of 1; 24 h**	7.5	Allyl alcohol	38.9	7.6	6.1	11 (*S*)	-	11.0	36.4
	7.5	AMA-1	49.9	9.3	8.2	6 (*S*)	-	6.6	25.9
	7.0	Cysteine	55.6	10.4	9.3	6 (*S*)	-	15.5	9.3
	7.5	3-Methylbutan-2-one	26.5	1.2	12.1	82 (*R*)	42.6	-	17.5
	7.5	4-Methylpentan-2-one	23.5	14.0	13.7	1 (*S*)	34.1	-	14.6
	8.0	Ethyl chloroacetate	45.9	4.5	3.3	15 (*S*)	-	6.6	39.7

## Data Availability

Not applicable.

## Sample Availability

Samples of the compounds (S)-2 (90% ee), (R)-2 (90% ee) are available from the authors.
